# Further Illustrations of the Ulcerative Process

**Published:** 1836-04-01

**Authors:** 


### ON ULCERATION OF THE CARTILAGES OF JOINTS.

FURTHER REMARKS ON THE ULCERATIVE PROCESS. By *C. Aston Key*, Surgeon to Guy's Hospital, and Lecturer on Surgery.

[Medico-Chirurgical Transactions, Vol. XIX.—Continued.]

OUR readers may remember, that in a former number of this Journal we brought under their notice a paper from the pen of Mr. Key, on the mode in which ulceration takes place in joints. We took occasion to differ from that able surgeon, for whom none can entertain more respect than ourselves, and to oppose opinions that we conceived to be hypothetical. Mr. Key is too much of the man of science not to own that on scientific subjects free discussion is highly advantageous. Whatever may be the condition of the disputants, truth is always served by the free examination of opinions. To Mr. Key, then, we feel that no apology is due, for his object, the elucidation of the vital process of ulceration, will be equally promoted, whether his peculiar views prove erroneous or correct.

The see-saw of speculative doctrines has frequently attracted observation. The theory of one age, abandoned in the next, has been revived in the succeeding, and few notions in the world of medicine have been buried so deep, that scientific resurrection-men have not been enabled to exhume them.

In the paper before us Mr. Key has dug up the exploded theory of ulceration, and presents it in new attire and a more seductive form. He endeavours to prove that ulceration is not a process of absorption, as is now commonly believed, but an actual degeneration and resolution of the tissues—the ancient doctrine which was thought to be annihilated by the observations and the reasonings of Hunter.

Mr. Key's arguments for this present heterodoxy seem when closely sifted to be these:—

I. The first step which Nature takes in forming an abscess, is a deposit of the solid or fibrinous part of the blood, either mixed with or free from the red particles, according as the action is acute or chronic. This solid deposit remains for a certain period unchanged, but at length becomes softer in consistence, and towards the centre exhibits all the characters of completely formed pus. The cavity thus formed either increases in size by the same process, or the sides of the cyst become organized, and secrete pus. Thus the fluid in an abscess at first is the product of a softening process; afterwards it is a secretion from the vessels of the walls of the cyst.

Supposing these statements strictly correct, they would go to support the views of Mr. Key. But we feel inclined to doubt whether the preceding can fairly be regarded as a correct expression of the ordinary progress of an abscess. Supposing, however, the fact to be accurate, how is the central lymph converted into pus? We cannot conceive any action, independent of the vital influence of the vessels, unless it be an action analogous to putrefaction. Does lymph out of the body resolve itself into globular pus? Is it not strange, that a solid removed from the operation of the vessels should spontaneously resolve itself into a fluid, exhibiting no more marks of decomposition than the lymph of which it is the debris, and precisely similar to the secretion from living vessels themselves? These are some of the difficulties that attend the explanation of the fact stated by our author, supposing that fact to have been observed correctly.

II. In many diseases, proceeds Mr. Key, the softening extends not only to fibrinous or tubercular deposits, but also to the organized tissues of the body; and under its influence large portions of vascular tissue become broken down and mixed with the purulent depôt. In the red hepatization of the lungs under pneumonia, the cells of the organ are infiltrated with a solid deposit in sufficient quantity to render the lung solid, but still allowing the natural vascular tissue to be distinguished. In the more advanced stage of pneumonia, or the grey hepatization, the vascular tissue of the lung is no longer discernible; it is blended with the fibrinous deposit, and presents a uniform greyish solid mass. If time be allowed for the deposit to soften down, the natural texture of the lung is found to have lost its consistence, and breaks down under slight pressure; and in many instances appears to be resolved into a puriform mass. The same train of actions may be observed in the chronic tubercular disease of the lung, and in the strumous disease of absorbent glands. In a scrofulous gland, the deposit of tubercular matter, the softening process, the gradual blending of the natural tissue of the gland with the scrofulous mass until it can no longer be distinguished, can each be clearly followed, until the whole is converted into a mass of pus, here and there intermixed with the debris of the original glandular structure.

Here, again, much must rest on the absolute accuracy of the observation. If Mr. Key is right in supposing, that the centre of an *unorganized* fibrinous deposite softens into pus, without the operation of living vessels, that is sufficient to settle the question. But all turns, as we have said, on the absolute accuracy of the observation. Take the case of tubercle cited by our author. A small deposit is contained in a cyst, which no doubt is organized. Either the whole cyst excites irritation and suppuration around it, and is expelled, or the contents of the cyst soften, the cyst ulcerates, and the contents are expectorated. The latter is usually the case. In this instance, it is much more likely that a change should be wrought by a modification of exhalation and absorption from the interior of the cyst, than by simple resolution.

III. "Ulceration is a process analogous to the softening attending suppuration; it is a degeneration of tissue, a change in the affinities existing between its component parts, by which it becomes changed from a solid organized texture to a fluid inorganic mass. It differs from gangrene in being a vital action; while gangrene, by at once producing death in a part, prevents any such change taking place. In gangrene, the supply of blood to the part altogether ceases, while the integrity of tissue is preserved; under ulceration, the circulation in the vessels continues during the action, and the part still belongs to the living mass, and remains under the influence of vital action until its separation is completed.

Such disintegration of organized tissues, and their conversion into their original elements, is consistent with the usual operations of nature; and for animal structures to resume their former elementary state, and again to become the fluid of which they were originally composed, is by no means an unnatural transition."

"Ulcers of all kinds, if closely watched in their formation, clearly exhibit the breaking up of the tissue, and its gradual conversion into pus. In the earliest stage of an ulcer, before the vesicle has burst or the skin given way, the extent of substance lost is always compensated for by the amount of fluid formed. In its progress, when rapid, and when a large portion of structure is quickly destroyed by ulceration, a corresponding quantity of pus may be always seen to occupy its place; and when the action is more chronic, as in fibrous structures, the debris of the tissue can be seen mixed with the purulent fluid." 142.

The whole of the argument palpably rests on this proposition—that the tissue resolves itself into something like its constituent elements. But is pus any thing resembling the constituent element of *all* tissues? The pus from an ulcerating bone—a muscle—the skin, differs, cæteris paribus, in no material respect; yet the elements of those respective tissues display some very essential distinctions. What analogy is there between ordinary ulceration, and the softening in the centre of a fibrinous deposite in phlegmon? It is too remote to present itself to us. To say that such disintegration of organized tissues, and their conversion into their original elements, is consistent with the usual operations of nature—is to propose an inapplicable truism; for Mr. Key must first prove that the pus *is* the original element of bodies. The chemists will tell him it is a compound albuminous fluid, as compound as many employed in the economy, and more composite than some. When Nature resolves the tissues into their original elements, she never converts them into pus. Her operations, when left unrestricted, and watched in the dissecting-room, end in the production of substances, and the evolution of elementary components widely different from that.

"The supposition (continues Mr. Key) that nature carries into the circulating system noxious matters and poisoned tissues, which can be more effectually and more safely got rid of by other means, is carrying analogy beyond the bounds of probability. But if the formation of an ulcer be an act of absorption the parts that are removed in the formation of a chancre are so disposed of; the absorbents, in forming a chancre, carry into the system tissues tainted by the venereal poison, and must therefore in every instance contaminate the whole mass of circulating fluids. That an action so deleterious to the system should uniformly accompany the formation of every venereal sore, is highly improbable; and if it were so, a bubo ought to be one of the earliest accompaniments of chancre. But during the ulcerative stage of chancre, the glands in the groin usually remain free from infection: it is when the ulcerative stage is at an end, that the gland enlarges and bubo forms." 141.

Monstrous as the supposition in question may appear to Mr. Key, it is undoubtedly too true. Nature established absorbents for the wise and necessary purpose of renovating the structure of the body. If deleterious substances come in the way of those absorbents, their functions are not abruptly nullified, because an amiable and an ingenious physiologist imagines that they should be so. Put woorara in the cellular tissue, or introduce into it arsenic, or the vaccine matter, or the small-pox virus, and observe whether the absorbents refuse to do the work, which Mr. Key thinks it beyond the bounds of probability that they should do. Nature acts not by partial but by general laws; and those laws are not drilled through and through by exceptions. Absorption is the rule; and, whether it be a worn-out muscle or a morbid poison, absorption more or less takes place. If to that absorption we owe the secondary effects of lues—to it we owe, also, the preservation of life by inoculation and by vaccination. There is evil, no doubt, but the balance is immeasurably on the side of good; and when Mr. Key can point out any general laws of nature from which individual hardship does not spring, then, but not before, we will admit that there is *some* force in his argument.

If Mr. Key had selected an illustration that would prove against himself, he could not have taken one more mal-à-propos than syphilis. He seems to think it absurd to imagine, that the absorbents forming a chancre must, in *every* case, taint the system. No doubt they must; and we do not doubt that they do. The majority of patients, under such circumstances, would probably have secondary symptoms of that taint, were there no means of counteracting it. Aye, but (replies Mr. Key) all do not have those secondary symptoms—therefore the virus is not, in all, carried into the system. The fallacy of this argument is easily shewn. All who expose themselves to marsh miasma do not get marsh fever—all who are vaccinated do not get the cow-pox—all who are exposed to small-pox are not, and would not, be affected with it. Mr. Key, the great advocate of natural salutary efforts, would appear to overlook one very certain and very benevolent provision—that a certain number only of individuals are susceptible of the influence of any disease, and especially of morbid poisons. Some persons seem capable of resisting the action of the latter altogether—others are affected by them only in a modified degree. Some will not exhibit the cow-pox vesicle at all—others display an imperfectly-formed one. Some persons are not liable to syphilitic primary sores—others only have primary sores, and are not affected by secondary symptoms—the majority, perhaps, are prone to both.

We do not see to what Mr. Key's remarks on bubo tend, unless it be to shew that absorption has nothing to do with it. If it has not, we leave to Mr. Key to give us *his* theory of secondary symptoms. We would engage, to use a legal phrase, to drive a coach and six through any explanation which rejects the active operation of an absorbing process.

Mr. Key cites caries of the teeth as "a familiar example of ulceration." Yet Mr. Key must be aware that, by many, this has been regarded, not as ulceration, but as gangrene. Mr. Bell, for instance, repudiates the idea of caries, and deems it mortification of the tooth. Be that as it may, we do not see the gist of Mr. Key's remarks on this head, nor how the caries of the tooth, if it be such, supports his view of ulceration. For he concludes rightly, "that it is a living action, as no such change has been observed in a tooth after its removal from the body. Dead teeth do not decay." But, if ulceration be only disintegration, why should not such a change occur in a dead tooth? Resolution into its constituent elements is more likely to happen in dead tissues, than absorption is.

IV. "It is difficult to explain or to reconcile the action of some remedies with the supposition of absorption being concerned in ulceration. One of the most powerful remedies that we possess, and that exerts a remarkable control over the ulcerative process, is iodine. The most active phagedænic ulcers, that threaten the destruction of parts, are often found to yield in a surprising manner to the influence of this medicine, and to put on a healthy granulating appearance. And yet iodine is thought to act powerfully in increasing the action of the absorbents; tumours of considerable size often yielding to its action and becoming absorbed. How is it, that in the healing of an ulcer it checks and puts a stop to the absorbing process, and in the case of a tumour quickens the action of the absorbents and gets rid of the mass? Here are two actions of a remedy opposed to each other, and inexplicable so long as ulceration is regarded in the light of absorption. In the same person, and often on the same limb, we see the beneficial effects of iodine in the arrest of a spreading cachectic ulcer, and the absorption of a venereal node or periosteal effusion; two actions that present a paradox to those who view ulceration as effected through the agency of the absorbents. But the action of this and other remedies that arrest the progress of ulceration, is effected through another medium than the absorbents; it would seem to depend on some additional nervous or vital power imparted to the ulcerating surface, by which its vital energies are reinforced, and which enabled it to resist those repulsive forces that tend to disorganize it." 144.

If Mr. Key could prove that iodine was a great promoter of absorption, and at the same time one of the most efficient agents in checking rapid ulcerative action, we would admit that there was plausibility in this argument. But we differ toto cœlo from Mr. Key in his very premises. We deny that iodine is an efficient remedy in cases of phagedænic ulceration. Nay we will go farther, and affirm as a general fact that iodine is one of the very worst remedies that could be administered in phagedæna. Our readers must observe that phægedenic ulceration may occur in circumstances diametrically opposite—in a state of system requiring depletion—and in a state of depression and extreme irritability. The former, so far as we have seen, is the exception—the latter is the rule. It is possible, that in the former, iodine may now and then be beneficial—we are certain that, in the latter, opium, the volatile alkali, stimuli, are the remedies to which the surgeon must trust. These are the remedies which build up, rather than pull down, and as reasoning would suggest their use, experience justifies it. On this point we can speak with the positiveness of careful and extensive observation, and here on the premises of the argument we are opposed to our author.

Let us turn to the application of the preceding arguments. It seems when compressed to amount to this—that in cases of disease of an articulation, when we find the synovial membrane vascular or a false membrane actually developed, and the cartilage partially dissolved, we should conclude, that the vis medicatrix naturæ developed the membrane, in order that *it* might absorb the cartilage, and prevent the occurrence of ulceration. Such appears to us, so far as we are enabled to appreciate it, the theory of Mr. Key. But we will subjoin his ipsissima verba, in order that we may not mislead our readers.

"The purport of my former observations on this subject, was to illustrate the mode in which nature effects the absorption of articular cartilage in some forms of disease, but especially in that chronic form of action usually regarded as strumous. My present object is to point out the difference between this mode of removing the cartilage, and the ulcerative or destructive process.

The distinction is one that cannot fail to strike the pathologist. The one is a repairing process, established with a view to the ultimate anchylosis of the joint, and by an efficient provision to prevent an inflammatory process that would otherwise end in ulceration and suppuration. A membrane is gradually developed, by the agency of which the cartilage is absorbed, and which afterwards becomes the medium of anchylosis; thus the destruction of the joint is often prevented.

But ulceration of the cartilage is effected in the same manner as an ulcer is formed in soft parts; it is a destructive action that sooner or later is followed by suppuration of the joint. It commences in the structure of the cartilage itself, which, no longer under the influence of those forces that unite its integral parts, breaks up and becomes converted into a purulent mass, which mixing with the synovia of the joint, irritates the synovial membrane to inflammation, and ultimately to suppuration and ulceration." 146.

It must be clear to any one accustomed to exact reasoning, that to render the hypothesis before us probable, two things must be established:—first, the essential difference between ulceration and absorption—secondly, the fact, that such absorption is calculated to prevent ulceration. We will not go into the discussion at length, but will simply observe, that we are called on to admit a great deal that is not proved, and some things that are not probable. It is not proved that absorption and ulceration do differ—it is not probable that Nature would set up an inflammation to prevent an inflammation, and go by a roundabout process to do that which she could, were such her intention, effect much more simply and more efficiently.

Without entering the lists for exact reasoning in medicine, we would remark, that the majority of modern pathologists would adopt a simple and intelligible view of these states of absorption, and of destructive ulceration. They would be content to suppose that in the former case the disease was slighter, and in the latter more severe: and that from mere absorption of the cartilage, with very slight inflammatory action, up to destructive ulceration of the same tissue, with suppuration in the joint, and caries of the bone, there extends a pathological chain, the links of which are sufficiently discernible.

Mr. Key in conclusion appeals to time as the best, the only test of truth. He is right. A few years will set all in the proper point of view, and if Mr. Key's hypothesis is really correct, our criticisms will assuredly be pointless. Yet, to the best of our judgment, they are sound, and certainly they are the offspring of conviction.

